# Clinicopathologic Characteristics and Prognosis of ERBB2-Low Breast Cancer Among Patients in the National Cancer Database

**DOI:** 10.1001/jamaoncol.2022.7476

**Published:** 2023-02-23

**Authors:** Daniel S. Peiffer, Fangyuan Zhao, Nan Chen, Olwen M. Hahn, Rita Nanda, Olufunmilayo I. Olopade, Dezheng Huo, Frederick M. Howard

**Affiliations:** 1 Department of Medicine, University of Chicago, Chicago, Illinois; 2 Department of Public Health Sciences, University of Chicago, Chicago, Illinois

## Abstract

**Importance:**

Given conflicting results regarding the prognosis of erb-b2 receptor tyrosine kinase 2 (ERBB2; formerly HER2 or HER2/neu)–low breast cancer, a large-scale, nationally applicable comparison of ERBB2-low vs ERBB2-negative breast cancer is needed.

**Objective:**

To investigate whether ERBB2-low breast cancer is a clinically distinct subtype in terms of epidemiological characteristics, prognosis, and response to neoadjuvant chemotherapy.

**Design/Participants/Setting:**

This retrospective cohort study was conducted using the National Cancer Database, including 1 136 016 patients in the US diagnosed with invasive breast cancer from January 1, 2010, to December 31, 2019, who had ERBB2-negative disease and had immunohistochemistry results available. ERBB2-low tumors were classified as having an immunohistochemistry score of 1+, or 2+ with a negative in situ hybridization test. Data were analyzed from November 1, 2021, through November 30, 2022.

**Exposures:**

Standard therapy according to routine clinical practice.

**Main Outcomes and Measures:**

The primary outcomes were overall survival (OS), reported as adjusted hazard ratios (aHRs), and pathologic complete response, reported as adjusted odds ratios (aORs), for ERBB2-negative vs ERBB2-low breast cancer, controlling for age, sex, race and ethnicity, Charlson-Deyo Comorbidity Index score, treatment facility type, tumor grade, tumor histology, hormone receptor status, and cancer stage.

**Results:**

The study identified 1 136 016 patients (mean [SD] age, 62.4 [13.1] years; 99.1% female; 78.6% non-Hispanic White), of whom 392 246 (34.5%) were diagnosed with ERBB2-negative and 743 770 (65.5%) with ERBB2-low breast cancer. The mean (SD) age of the ERBB2-negative group was 62.1 (13.2) years and 62.5 (13.0) years for the ERBB2-low group. Higher estrogen receptor expression was associated with increased rates of ERBB2-low disease (aOR, 1.15 per 10% increase). Compared with non-Hispanic White patients, of whom 66.1% were diagnosed with ERBB2-low breast cancer, fewer non-Hispanic Black (62.8%) and Hispanic (61.0%) patients had ERBB2-low disease, although in non-Hispanic Black patients this was mediated by differences in rates of triple-negative disease and other confounders. A slightly lower rate of pathologic complete response was seen in patients with ERBB2-low disease vs patients with ERBB2-negative disease on multivariable analysis (aOR, 0.89; 95% CI, 0.86-0.92; *P* < .001). ERBB2-low status was also associated with small improvements in OS for stage III (aHR, 0.92; 95% CI, 0.89-0.96; *P* < .001) and stage IV (aHR, 0.91; 95% CI, 0.87-0.96; *P* < .001) triple-negative breast cancer, although this amounted to only a 2.0% (stage III) and 0.4% (stage IV) increase in 5-year OS.

**Conclusions and Relevance:**

This large-scale retrospective cohort analysis found minimal prognostic differences between ERBB2-low and ERBB2-negative breast cancer. These findings suggest that, moving forward, outcomes in ERBB2-low breast cancer will be driven by ERBB2-directed antibody-drug conjugates, rather than intrinsic differences in biological characteristics associated with low-level ERBB2 expression. These findings do not support the classification of ERBB2-low breast cancer as a unique disease entity.

## Key Points

**Question:**

Do the demographics, clinicopathologic characteristics, and prognosis differ between breast cancers with no erb-b2 receptor tyrosine kinase 2 (ERBB2; formerly HER2 or HER2/neu) expression (ERBB2 negative) and those with low-level ERBB2 expression (ERBB2 low)?

**Findings:**

In this cohort study of 1 136 016 patients from the National Cancer Database in the US, the proportions of ERBB2-low breast cancer were slightly lower among Hispanic and non-Hispanic Black patients compared with non-Hispanic White patients. ERBB2-low status was associated with slightly improved overall survival (≤2% difference at 5 years) compared with ERBB2-negative cancer.

**Meaning:**

The findings of this study suggest that treatment response and long-term outcomes may be similar in ERBB2-low and ERBB2-negative cancers and do not support the classification of ERBB2-low breast cancer as a unique disease entity.

## Teaser

This cohort study uses National Cancer Database data to investigate whether breast cancer with low ERBB2 expression is a clinically distinct subtype compared with breast cancer with no ERBB2 expression, with distinct epidemiological characteristics, prognosis, and response to neoadjuvant chemotherapy.

## Introduction

Erb-b2 receptor tyrosine kinase 2 (ERBB2; formerly HER2/neu)–positive breast cancer was first defined as breast cancer with an amplification of *ERBB2* leading to ERBB2 overexpression.^1^ ERBB2 is clinically assessed with immunohistochemistry (IHC), with expression scored from 0 to 3+, and/or with in situ hybridization (ISH), which can detect *ERBB2* amplification. ERBB2-positive cancers are currently defined as those with high expression (3+) via IHC or *ERBB2* amplification via ISH. The precise classification of ERBB2-positive breast cancer has evolved to align with tumors predicted to respond to ERBB2-targeted therapies such as trastuzumab, constituting about 15% of all breast cancers.^2^ Although approximately 50% to 60% of all breast cancers exhibit a low level of ERBB2 expression via IHC, cancers with low expression benefit from standard ERBB2-targeted therapies.^3,4,5,6,7^ The paradigm of ERBB2 classification has recently shifted with the development of antibody-drug conjugates such as trastuzumab-deruxtecan (T-DXd), which has demonstrated efficacy in patients with ERBB2-low breast cancer, defined as 1+ ERBB2 expression via IHC or 2+ with negative ISH.^8,9^ This has led to renewed interest in the subgroup of ERBB2-low breast cancer because greater understanding of the biological characteristics of this patient population could yield additional therapeutic approaches.

However, ERBB2-low tumors are heterogeneous, consisting of both hormone receptor–positive and triple-negative breast cancer (TNBC).^2,5,6^ Multiple groups have undertaken studies to define ERBB2-low tumors in terms of epidemiological and clinicopathologic parameters; however, this has yielded conflicting results such as rates of ERBB2-low status among TNBC ranging from 40.1%^10^ to 66.3%.^11^ Moreover, there are conflicting reports of ERBB2-low status being associated with a negative,^5,10,11^ neutral,^12,13^ or positive prognosis.^14,15^ Previous studies have had limited geographical distribution and/or relatively small sample sizes in the cohort analyzed, limiting the power to discern a prognostic difference between ERBB2-low and ERBB2-negative (ERBB2 expression scored as 0 via IHC) breast cancer and the generalizability of the results. Furthermore, given the known racial and ethnic disparities in breast cancer prognosis, it is important to describe the epidemiological characteristics of ERBB2-low breast cancer to understand the potential association with novel antibody-drug conjugates such as T-DXd on breast cancer disparities. Our aim was to investigate whether ERBB2-low breast cancer is a clinically distinct subtype in terms of epidemiological characteristics, prognosis, and response to neoadjuvant chemotherapy.

## Methods

### Study Design and Data Source

This retrospective cohort study examined and compared the epidemiological differences and prognostic significance of ERBB2-low vs ERBB2-negative breast cancer. We abstracted patient data from the National Cancer Database (NCDB), the largest cancer registry in the world, which includes data from approximately 70% of new invasive cancer diagnoses in the US.^16^ The data were analyzed from November 1, 2021, through November 30, 2022. This study was deemed exempt from review by the institutional review board at the University of Chicago per 45 CFR part 46 of the Human Health Services regulations for human subjects research, which defines the criteria for secondary research for which consent is not required. No informed consent was obtained because NCDB data are deidentified. The data collected by the American Cancer Society/Commission on Cancer (ACS/CoC) for the NCDB are collected passively through cancer registries without informed consent collected from patients. The ACS/CoC and participating hospitals exempted the requirement of informed consent. This study followed the Strengthening the Reporting of Observational Studies in Epidemiology (STROBE) guideline for cohort studies.^17^

### Study Population and Covariates

Patients from the NCDB who were diagnosed with invasive breast cancer from January 1, 2010, to December 31, 2019, that was not classified as ERBB2 positive and who had ERBB2 IHC results available were included. ERBB2-low cases were defined as those with an IHC score of 1+, or 2+ with negative ISH results, and ERBB2-negative cases were defined as those with an IHC score of 0. We excluded patients with an ERBB2 IHC score of 2+ disease who did not have documented negative ISH results. We only included cases of invasive disease and excluded patients classified as stage 0 or with unknown stage.

The following epidemiological and clinicopathologic parameters were analyzed and compared between the ERBB2-low and ERBB2-negative groups: age at diagnosis; race and ethnicity; treatment facility type; tumor, nodal, and overall stage; tumor histology and grade; site of metastasis (including bone, brain, liver, and lung); quantitative estrogen receptor (ER) and progesterone receptor (PR) status; Ki-67 status determined via IHC, Oncotype DX (Exact Sciences Corp) multigene assay score; *ERBB2* copy number and/or *ERBB2*/centromeric region of chromosome 17 (*CEP17*) ratio determined via ISH; receipt of chemotherapy and/or hormonal therapy; and survival status. Race and ethnicity were included in this analysis as part of a comprehensive assessment of the relationship of demographic factors and ERBB2-low status. The NCDB reported that the race and ethnicity information is collected from cancer program registries from patient self-report and medical/billing records. The race and ethnicity categories included Asian or Pacific Islander, Hispanic, Native American, Non-Hispanic Black, Non-Hispanic White, and other. Other is a subcategory listed in the NCDB and represents patients who were coded as Other by the local cancer registry. No explicitly defined racial or ethnic subgroup categorized by the NCDB was collapsed into Other. The TNM tumor staging was based on pathological stage if available, except in patients who received neoadjuvant chemotherapy (defined as receipt of chemotherapy at least 30 days prior to surgery), in which case clinical stage was used. Pathologic complete response (pCR) status was also recorded for patients who received neoadjuvant chemotherapy. As with all variables used in our analysis, race and ethnicity were abstracted by certified cancer registrars from the medical records for inclusion in the NCDB. Missing covariates were inferred with multiple imputation by chained equations.^18^

### Statistical Analysis

We first performed descriptive statistics to compare clinicopathologic and epidemiological features between ERBB2-low and ERBB2-negative subgroups, using a χ^2^ test to compare categorical variables and an unpaired *t* test to compare continuous variables. A multivariable logistic regression analysis was used to quantify the independent association of these features with low-level ERBB2 expression, and adjusted odds ratios (aORs) were reported with significance and 95% CIs computed via Wald statistic. A similar logistic regression model was formulated to examine the association of ERBB2-low status with sites of metastatic disease. Overall survival (OS) was defined as the time from date of diagnosis to the last follow-up or death. Kaplan-Meier curves for OS were calculated for ERBB2-low vs ERBB2-negative breast cancer, stratified by receptor subtype and stage group. Multivariable Cox proportional hazards regression models were fit to examine the independent prognostic value of ERBB2-low breast cancer. For patients who received neoadjuvant therapy, a multivariable logistic regression analysis was used to examine the association of ERBB2-low status with pCR. All multivariable analyses controlled for age, sex, race and ethnicity, Charlson-Deyo Comorbidity Index score, treatment facility type, tumor grade and histology, and quantitative ER and PR status (except for analyses in the TNBC subgroup). Models for survival in the entire cohort and prediction of ERBB2-low status included overall stage group, and models for pCR included T and N stage groups. Statistical analysis was performed using Python, version 3.7.5 (Python Software Foundation), using the statsmodels, version 0.13.2, and lifelines, version 0.27.1. The code used for data extraction and statistical testing is publicly available.^19^ All statistical tests were 2-sided with a significance threshold of *P* < .05.

## Results

### Epidemiological and Clinicopathologic Features of Patients With ERBB2-Low Breast Cancer

We identified 1 136 016 patients (mean [SD] age, 62.4 [13.1] years; 99.1 female; 78.6% non-Hispanic White), comprising 392 246 patients (34.5%) diagnosed with ERBB-negative and 743 770 (65.5%) with ERBB2-low breast cancer who met the criteria for study inclusion (Table 1 and eFigure 1 in Supplement 1). As shown in Table 1 and eTable 1 in Supplement 1, 99.1% of patients were female, and the mean (SD) age at primary diagnosis was 62.1 (13.2) years for the ERBB2-negative group and 62.5 (13.0) years for the ERBB2-low group.

**Table 1.** Baseline Patient Demographics and Clinicopathologic Characteristics

Characteristic	Participants, %^a^			*P* value^b^
	Total (N = 1 136 016)	ERBB2-negative status (n = 392 246)	ERBB2-low status (n = 743 770)	
Age, mean (SD), y				
<40	4.1	4.5	3.9	<.001
40-49	13.9	14.2	13.8	
50-59	22.5	22.5	22.5	
60-69	28.8	28.4	28.9	
70-79	20.9	20.8	20.9	
≥80	9.8	9.6	10.0	
Sex				
Female	99.1	99.3	99.0	<.001
Male	0.9	0.7	1.0	
Race and ethnicity (9325^c^)				
Asian	3.7	3.6	3.7	<.001
Hispanic	5.6	6.3	5.2	
Native American	0.3	0.2	0.3	
Non-Hispanic Black	11.3	12.1	10.8	
Non-Hispanic White	78.6	77.1	79.5	
Other^d^	0.5	0.6	0.5	
Facility type (46 482^c^)				
Academic or research	30.6	34.1	28.8	<.001
Community cancer program	7.2	7.0	7.3	
Comprehensive community cancer program	41.4	39.0	42.6	
Integrated network cancer program	20.8	19.9	21.3	
Charlson-Deyo Comorbidity Index score				
0	82.0	82.2	81.9	.003
≥1	18.0	17.8	18.1	
Grade (48 845^c^)				
1	26.1	24.4	27.0	<.001
2	47.3	44.6	48.7	
3	26.6	31.0	24.3	
Tumor histological characteristics				
Ductal	77.7	75.5	78.9	<.001
Ductal and lobular	5.3	5.2	5.3	
Inflammatory	0.2	0.2	0.1	
Lobular	11.7	12.4	11.3	
Medullary	0.1	0.2	0.1	
Metaplastic	0.5	1.0	0.3	
Mucinous	2.0	2.7	1.6	
Paget disease	0.1	0	0.1	
Papillary	0.3	0.3	0.3	
Sarcoma	0	0	0	
Tubular	0.6	0.5	0.6	
Other	1.6	2.0	1.4	
T stage (96 912^c^)				
0	0.3	0.3	0.3	<.001
1	65.2	64.3	65.7	
2	27.3	27.8	27.1	
3	5.0	5.4	4.8	
4	2.1	2.2	2.1	
N stage (134 296^c^)				
0	72.2	72.8	71.9	<.001
1	21.6	21.0	21.9	
2	4.0	3.9	4.1	
3	2.2	2.3	2.2	
Stage				
I	59.7	58.8	60.2	<.001
II	27.2	27.5	27.1	
III	8.7	9.1	8.5	
IV	4.4	4.6	4.3	
Bone metastasis at diagnosis (11 995^c^)				
No	97.7	97.7	97.7	.13
Yes	2.3	2.3	2.3	
Brain metastasis at diagnosis (12 354^c^)				
No	99.8	99.7	99.8	<.001
Yes	0.2	0.3	0.2	
Liver metastasis at diagnosis (12 270^c^)				
No	99.3	99.3	99.4	<.001
Yes	0.7	0.7	0.6	
Lung metastasis at diagnosis (12 403^c^)				
No	99.0	99.0	99.1	.001
Yes	1.0	1.0	0.9	
*ERBB2*/*CEP17* ratio (793 991), mean (SD)	1.3 (1.3)	1.3 (2.2)	1.3 (1.0)	.73
*ERBB2* copies (1 050 922^c^), mean (SD)	2.7 (4.2)	2.4 (4.7)	2.8 (4.0)	<.001
Receptor status (7217^c^)				
ER positive, PR positive	75.8	69.2	79.3	<.001
ER positive, PR negative	9.7	9.8	9.6	
ER negative, PR positive	0.8	1.1	0.7	
ER negative, PR negative	13.6	19.9	10.3	
Percentage ER positive (860 499^c^), mean (SD)	78.1 (35.2)	70.8 (40.2)	82.5 (31.1)	<.001
Percentage PR positive (860 403^c^), mean (SD)	53.9 (40.8)	49.8 (42.2)	56.4 (39.7)	<.001
Percentage Ki67 positive (997 909^c^), mean (SD)	24.6 (26.8)	27.6 (30.1)	22.8 (24.5)	<.001
Oncotype Dx score (853 587^c^)				
High (≥26)	12.5	12.4	12.6	<.001
Intermediate (11-25)	62.5	61.6	62.9	
Low (0-10)	25.0	26.1	24.5	
Receipt of chemotherapy (13 635^c^)				
Yes	34.5	37.2	33.0	<.001
No	65.5	62.8	67.0	
Receipt of hormonal therapy (26 355^c^)				
Yes	73.3	67.4	76.4	<.001
No	26.7	32.6	23.6	

Abbreviations: *CEP17*, centromeric chromosome 17; ER, estrogen receptor; *ERBB2*, erb-b2 receptor tyrosine kinase 2; PR, progesterone receptor.

^a^ Percentages reflect the number of participants missing, as shown in the left column.

^b^ *P* values are listed for a χ^2^ test for categorical variables and a 2-sided *t* test for continuous variables.

^c^ Indicates number of patients for whom data were missing.

^d^ Other is a subcategory listed in the National Cancer Database (NCDB) and represents patients who were coded as such by the local cancer registry. No explicitly defined racial or ethnic subgroup categorized by the NCDB was collapsed into this category.

Compared with non-Hispanic White patients, of whom 66.1% had ERBB2-low breast cancer, Native American patients had a higher proportion of ERBB2-low disease (2270 [70.0%]), while the proportions were lower in non-Hispanic Black (62.8%) and Hispanic (61.0%) patients. The proportion of ERBB2-low diagnoses was lower at academic or research centers (61.8%) than at community cancer programs (66.5%), comprehensive community cancer programs (67.5%), or integrated network cancer programs (67.1%). Fewer ductal tumors were ERBB2 negative (33.6%) compared with lobular (36.8%) or mucinous (46.4%) tumors. Most metaplastic (64.0%) and medullary (50.2%) cancers and sarcomas (67.8%) were ERBB2 negative. A higher rate of ERBB2-low disease was seen in hormone receptor–positive cancers (TNBC, 51.5% for ERBB2-low compared with 58.6% for ER-negative, PR-positive cancers; 66.1% for ER-positive, PR-negative cancers; and 69.1% for ER-positive, PR-positive cancers). This was also reflected in the subset of patients with quantitative ER and PR testing results, which were higher in ERBB2-low (mean [SD], 82.5% [31.1%] ER expression, 56.4% [39.7%] PR expression) compared with ERBB2-negative (mean [SD], 70.8% [40.2%] ER expression, 49.8% [42.2%] PR expression) cancers. Among the high-grade tumors (ie, grade 3), only 59.8% were ERBB2 low, compared with 67.8% of intermediate- and low-grade tumors.

Given the potential confounding effects of clinicopathologic factors, we conducted a multivariable logistic regression analysis to examine the independent association of ERBB2-low status with age, sex, race and ethnicity, Charlson-Deyo Comorbidity Index score, treatment facility type, receptor status, stage, grade, and tumor histology (Table 2). Increased ER expression was associated with a higher likelihood of ERBB2-low status (aOR, 1.15 per 10% increase; 95% CI, 1.15-1.15; *P* < .001), but a lower likelihood of ERBB2-low was seen with increased PR expression (aOR, 0.95 per 10% increase; 95% CI, 0.95-0.95; *P* < .001). This may in part be associated with patients with weak ER or strong PR expression, who were less likely to have ERBB2-low breast cancer (eFigure 2 in Supplement 1). Most nonductal histological tumors were associated with lower proportions of ERBB2-low breast cancer than ductal cancers, including lobular (aOR, 0.73; 95% CI, 0.72-0.74; *P* < .001), mucinous (aOR, 0.51; 95% CI, 0.49-0.52; *P* < .001), and metaplastic (aOR, 0.53; 95% CI, 0.50-0.56; *P* < .001) cancers. Compared with non-Hispanic White patients, non-Hispanic Black (aOR, 1.01; 95% CI, 1.00-1.03; *P* = .06), and Asian or Pacific Islander patients (aOR, 1.02; 95% CI, 0.99-1.04; *P* = .15) had similar rates of ERBB2-low disease. Native American (aOR, 1.22; 95% CI, 1.13-1.32; *P* < .001) patients had higher proportions of ERBB2-low disease, whereas Hispanic patients had lower proportions (aOR, 0.85; 95% CI, 0.83-0.86; *P* < .001). In a separate multivariable logistic regression analysis to examine the association of sites of metastasis with ERBB2 expression in patients with stage IV disease, ERBB2-low status was associated with slightly lower rates of brain metastases (aOR, 0.88; 95% CI, 0.81-0.96; *P* < .001; eTable 2 in Supplement 1).

**Table 2.** Multivariable Analysis for Epidemiological and Demographic Factors Associated With ERBB2-Low Status^a^

Variable	ERBB2-low status, aOR (95% CI)	*P* value
Age, per 10 y	0.98 (0.98-0.98)	<.001
Sex		
Female	1 [Reference]	NA
Male	1.00 (0.98-1.03)	.68
Charlson-Deyo Comorbidity Index		
0	1 [Reference]	NA
≥1	1.01 (1.00-1.01)	.14
Facility type		
Community cancer program	1 [Reference]	NA
Comprehensive community cancer program	1.07 (1.05-1.09)	<.001
Academic or research	0.83 (0.81-0.84)	<.001
Integrated network cancer program	1.04 (1.02-1.06)	<.001
Receptor, per 10% expression		
Estrogen	1.15 (1.15-1.15)	<.001
Progesterone	0.95 (0.95-0.95)	<.001
Race and ethnicity		
Asian or Pacific Islander	1.02 (0.99-1.04)	.15
Hispanic	0.85 (0.83-0.86)	<.001
Native American	1.22 (1.13-1.32)	<.001
Non-Hispanic Black	1.01 (1.00-1.03)	.06
Non-Hispanic White	1 [Reference]	NA
Other^b^	0.84 (0.80-0.89)	<.001
Stage		
I	1 [Reference]	NA
II	1.11 (1.10-1.12)	<.001
III	1.12 (1.11-1.14)	<.001
IV	1.00 (0.98-1.02)	.91
Grade		
1	1 [Reference]	NA
2	1.02 (1.01-1.03)	<.001
3	1.04 (1.03-1.06)	<.001
Tumor histology		
Ductal	1 [Reference]	NA
Lobular	0.73 (0.72-0.74)	<.001
Ductal and lobular	0.85 (0.84-0.87)	<.001
Mucinous	0.51 (0.49-0.52)	<.001
Papillary	0.95 (0.88-1.03)	.23
Tubular	0.99 (0.94-1.05)	.75
Inflammatory	1.00 (0.91-1.11)	.99
Medullary	0.94 (0.85-1.04)	.25
Metaplastic	0.53 (0.50-0.56)	<.001
Paget disease	1.24 (1.04-1.48)	.02
Sarcoma	0.5 (0.38-0.67)	<.001
Other	0.85 (0.82-0.87)	<.001

Abbreviations: aOR, adjusted odds ratio; ERBB2, erb-b2 receptor tyrosine kinase 2; NA, not applicable.

^a^ Analysis performed with logistic regression in all 1 136 016 patients, with imputation for missing values.

^b^ Other is a subcategory listed in the National Cancer Database (NCDB) and represents patients who were coded as such by the local cancer registry. No explicitly defined racial or ethnic subgroup categorized by the NCDB was collapsed into this category.

### Prognosis of ERBB2-Negative vs ERBB2-Low Breast Cancer

Within our cohort, 109 588 patients received neoadjuvant chemotherapy, and 99 783 had pathological outcomes available in order to compare rates of pCR (eTable 3 in Supplement 1). Overall, of 39 688 ERBB2-negative patients, 9372 (23.6%) experienced pCR compared with 9812 (16.3%) of 60 095 ERBB2-low patients. In the total hormone receptor–positive subset, the pCR rate was 11.5% in ERBB2-negative and 8.9% in ERBB2-low patients. The difference was similar in the TNBC subset, with a pCR rate of 33.4% in ERBB2-negative and 30.2% in ERBB2-low patients. Similar results were found on multivariable analysis: ERBB2-low status was associated with a slightly reduced likelihood of pCR (aOR, 0.89; 95% CI, 0.86-0.92) compared with ERBB2-negative status; *P* < .001) (Table 3). A similar effect size was seen in patients with ERBB2 1+ (aOR, 0.89; 95% CI, 0.85-0.92; *P* < .001) and ERBB2 2+ (aOR, 0.89; 95% CI, 0.84-0.93; *P* < .001) status when separating ERBB2-low into these 2 groups. The effect size was also similar when analysis was repeated with just TNBC (aOR, 0.89; 95% CI, 0.85-0.93; *P* < .001) or just hormone receptor–positive cancers (aOR, 0.92; 95% CI, 0.86-0.98; *P* = .008).

**Table 3.** Multivariable Analysis Incorporating ERBB2 Expression for Probability of Pathologic Complete Response After Neoadjuvant Chemotherapy^a^

Variable	pCR, aOR (95% CI)	*P* value
ERBB2 via immunohistochemistry		
ERBB2 negative	1 [Reference]	NA
ERBB2 low	0.89 (0.86-0.92)	<.001
Age, per 10 y	0.85 (0.84-0.86)	<.001
Sex		
Females	1 [Reference]	NA
Males	0.99 (0.85-1.15)	.89
Charlson-Deyo Comorbidity Index		
0	1 [Reference]	NA
≥1	0.92 (0.89-0.96)	<.001
Facility type		
Community cancer program	1 [Reference]	NA
Comprehensive community cancer program	1.13 (1.04-1.24)	.004
Academic or research	1.15 (1.05-1.25)	.002
Integrated network cancer program	1.21 (1.11-1.33)	<.001
Expression, per 10% increase		
ER	0.91 (0.91-0.92)	<.001
PR	0.88 (0.87-0.89)	<.001
Race and ethnicity		
Asian or Pacific Islander	0.99 (0.91-1.08)	.86
Hispanic	0.98 (0.92-1.04)	.55
Native American	0.83 (0.62-1.12)	.23
Non-Hispanic Black	0.85 (0.82-0.89)	<.001
Non-Hispanic White	1 [Reference]	NA
Other^b^	0.96 (0.77-1.20)	.72
T stage		
1	1 [Reference]	NA
2	0.71 (0.68-0.74)	<.001
3	0.46 (0.43-0.49)	<.001
4	0.39 (0.36-0.43)	<.001
N stage		
0	1 [Reference]	NA
1	0.72 (0.7-0.75)	<.001
2	0.66 (0.62-0.72)	<.001
3	0.71 (0.65-0.77)	<.001
Grade		
1	1 [Reference]	NA
2	1.06 (0.94-1.2)	.35
3	2.1 (1.86-2.37)	<.001
Tumor histology		
Ductal	1 [Reference]	NA
Lobular	0.45 (0.39-0.52)	<.001
Ductal and lobular	0.54 (0.46-0.63)	<.001
Mucinous	0.45 (0.24-0.86)	.01
Papillary	1.14 (0.66-1.96)	.64
Tubular	0.51 (0.07-3.79)	.51
Inflammatory	0.69 (0.52-0.91)	.008
Medullary	1.25 (0.86-1.81)	.23
Metaplastic	0.24 (0.2-0.29)	<.001
Paget disease	0.57 (0.17-1.93)	.36
Sarcoma	0.19 (0.06-0.64)	.007
Other	1.29 (1.16-1.43)	<.001

Abbreviations: aOR, adjusted odds ratio; ER, estrogen receptor; ERBB2, erb-b2 receptor tyrosine kinase 2; NA, not applicable; pCR, pathologic complete response; PR, progesterone receptor.

^a^ Analysis performed with logistic regression in 99 784 patients receiving neoadjuvant chemotherapy, with imputation for missing values.

^b^ Other is a subcategory listed in the National Cancer Database (NCDB) and represents patients who were coded as such by the local cancer registry. No explicitly defined racial or ethnic subgroup categorized by the NCDB was collapsed into this category.

At the time of survival analysis, the median (IQR) follow-up was 54 (33-80) months with 831 645 (84.2%) patients with known survival status. On multivariable analysis of the entire cohort for whom survival was available (n = 987 934), ERBB2-low status was positively associated with survival, although the difference was minimal (aHR, 0.98; 95% CI, 0.97-0.99; *P* < .001). Given the possible interactions among ERBB2-low status, tumor stage, and receptor status, multivariable analysis was repeated separately by stage and receptor subtype (Figure). Associations were seen between ERBB2-low status and survival for stages II to IV TNBC and stages III to IV hormone receptor–positive cancer, although these differences were also small (Table 4). Associations with the lowest aHRs were seen for stage III TNBC (median OS difference, 6.5 months; aHR, 0.92; 95% CI, 0.89-0.96; *P* < .001) and stage IV TNBC (median OS difference, 1.7 months; aHR, 0.91; 95% CI, 0.87-0.96; *P* < .001), but this represented only a 2.0% (stage III) and 0.4% (stage IV) improvement in 5-year OS, respectively. Splitting ERBB2-low into ERBB2 2+ and 1+ was associated with a greater survival benefit with ERBB2 2+ for all subgroups of stage and receptor status (eTable 4 in Supplement 1).

**Figure.**
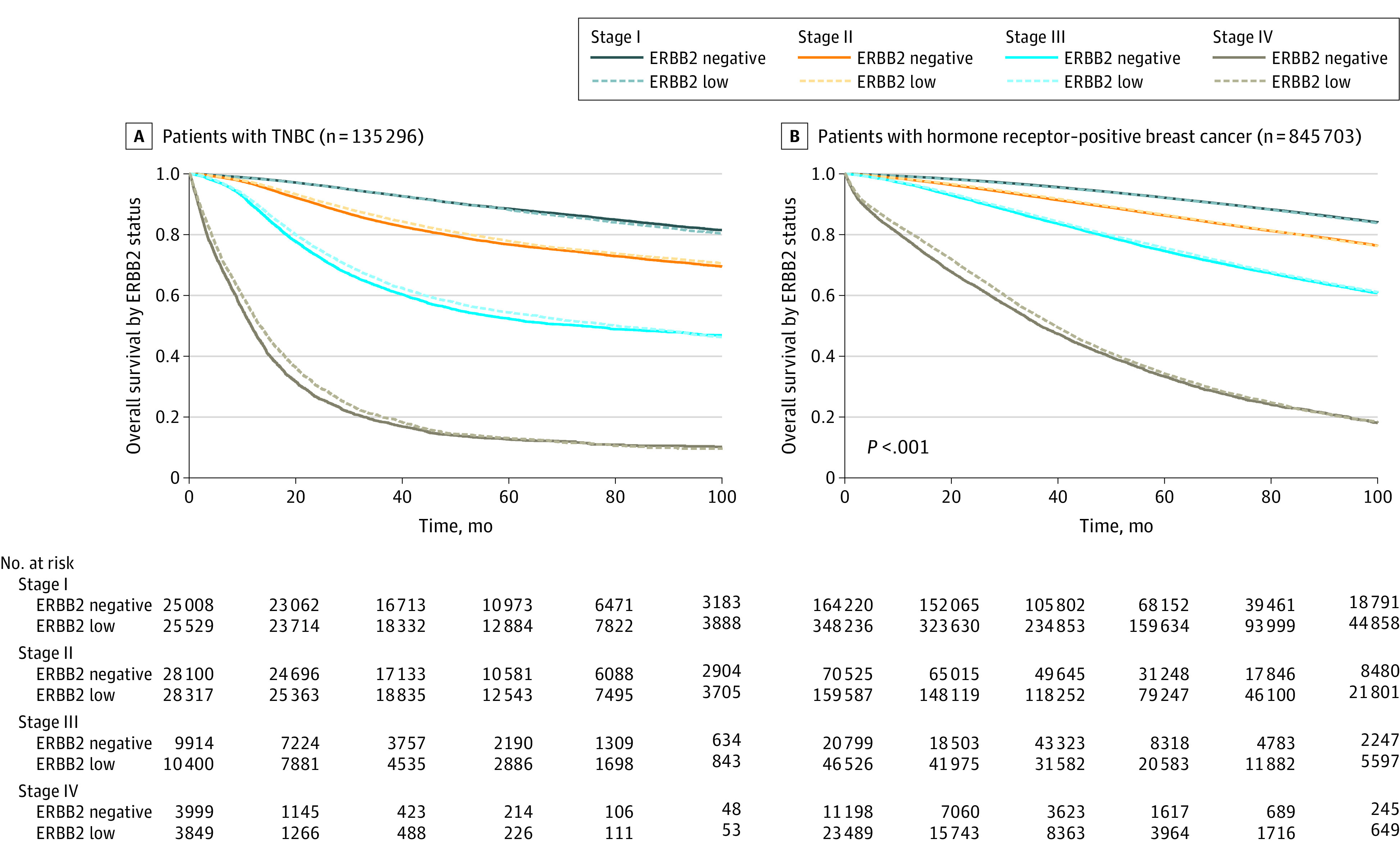
Kaplan-Meier Plots Showing Overall Survival by Erb-b2 Receptor Tyrosine Kinase 2 (ERBB2)–Low Status in Patients With Triple-Negative Breast Cancer (TNBC) and Hormone Receptor–Positive Breast Cancer Kaplan-Meier survival curves are illustrated by stage and hormone receptor status for ERBB2-low vs ERBB2-negative breast cancer.

**Table 4.** Overall Survival in ERBB2-Negative vs ERBB2-Low Cancers, by Cancer Subtype and Stage

Stage	Participants, No.		5-Year OS (95% CI)		Median (IQR) OS (95% CI)		aHR (95% CI)^a^	*P* value
	ERBB2-negative status	ERBB2-low status	ERBB2-negative status	ERBB2-low status	ERBB2-negative status	ERBB2-low status		
**Triple-negative breast cancer**								
I	25 056	25 568	88.5 (88.1-89.0)	88.2 (87.7-88.6)	NR	NR	1.01 (0.97-1.05)	.68
II	28 136	28 353	76.8 (76.2-77.3)	77.9 (77.3-78.4)	NR	NR	0.94 (0.91-0.97)	<.001
III	9920	10 410	52.4 (51.2-53.5)	54.4 (53.3-55.5)	73.8 (64.7-82.0)	80.3 (74.5-87.8)	0.92 (0.89-0.96)	<.001
IV	4003	3850	12.7 (11.4-13.8)	13.1 (11.7-14.2)	11.6 (11.2-12.1)	13.3 (12.7-14.0)	0.91 (0.87-0.96)	<.001
**Hormone receptor–positive breast cancer**								
I	164 484	348 786	92.1 (92.0-92.3)	92.1 (92.0-92.2)	NR	NR	1.01 (0.99-1.02)	.24
II	70 603	159 750	86.3 (86.0-86.6)	86.5 (86.4-86.7)	NR	NR	0.99 (0.97-1.01)	.36
III	20 811	46 549	74.6 (74.0-75.3)	75.6 (75.2-76.0)	131.6 (127.2-NR)	134.2 (130.3-NR)	0.97 (0.94-1.00)	.02
IV	11 211	23 509	33.4 (32.3-34.5)	34.3 (33.6-35.0)	36.7 (35.8-38.0)	39.5 (38.9-40.2)	0.96 (0.94-0.99)	.006

Abbreviations: aHR, adjusted hazard ratio; ERBB2, erb-b2 receptor tyrosine kinase 2; NR, not reached; OS, overall survival.

^a^ Adjusted hazard ratio is listed for a multivariable Cox proportional hazards regression model including age, sex, Charlson-Deyo Comorbidity Index score, facility type, race and ethnicity, grade, histological subtype, and quantitative estrogen and progesterone receptor status (in the hormone receptor–positive model), with imputation for missing values.

### Association of ERBB2-Low Status With ISH Results

It is known that there is great variability in stratifying the ERBB2-low from ERBB2-negative subtype by IHC.^21^ Thus, we assessed differences between ERBB2 copy number and *ERBB2*/*CEP17* ratio to examine if the results of ISH testing results differ between ERBB2-negative and ERBB2-low patients to aid in selecting patients for ERBB2-low–directed therapies (eFigure 3 in Supplement 1). Optimal cutoffs for copy number and ratio were selected (maximizing Youden’s index). The optimal *ERBB2*/*CEP17* ratio of greater than 1.09 was associated with a sensitivity of 76.6% for ERBB2-low disease and a specificity of 36.8%, while an *ERBB2* copy number of greater than 2.01 had a sensitivity of 61.1% and specificity of 58.2%. Performance characteristics at other cutoffs were also evaluated (eTable 5 in Supplement 1).

## Discussion

This cohort study is, to our knowledge, the first to use the NCDB in over 1 million patients diagnosed with ERBB2-low or ERBB2-negative breast cancer and reflects the geographic and ethnic distribution typically seen in the disease within the US.^22^ Similar to prior studies,^10,11^ we found that ERBB2-low status is more frequent in hormone receptor–positive than triple-negative disease. Previous studies^5,10^ have also reported a higher rate of ductal histology among ERBB2-low cases, but our study also found associations with other subtypes, such as less frequent ERBB2-low disease in metaplastic and medullary tumors. In contrast to prior work,^12^ ERBB2-low disease was associated with lower rates of brain metastasis. Our analysis also found slightly lower rates of ERBB2-low disease in non-Hispanic Black and Hispanic women. For non-Hispanic Black patients, this difference was mediated by other known clinicopathologic features such as higher proportions of TNBC,^23,24^ highlighting the complex interplay between low-level ERBB2 expression and patient ancestry that deserves further study.

Whether ERBB2-low breast cancer represents a unique subtype is controversial^20,25^; multiple studies have found associations of ERBB2-low status with response to therapy and prognosis. However, the size of these differences in our study and others^10,11^ was small and confounded by hormone receptor status. We found a 2.6% reduction in pCR rates in hormone receptor–positive ERBB2-low disease and a 3.2% reduction in pCR rates among TNBC ERBB2-low patients—these reductions persisted on multivariable analysis, likely due, in part, to the large sample size. These findings are in line with the 4.6% lower pCR rates in ERBB2-low TNBC seen by Tarantino et al^20^ or the approximately 6% lower rates of pCR in hormone receptor–positive ERBB2-low disease seen in multiple studies.^10,15^ However, the clinical significance of these differences is questionable, and the size of the differences was similar to a 10% increase in ER expression on multivariable analysis seen in our study (ie, the aOR for ERBB2-low vs ERBB2-negative was 0.89, similar to a 10% increase in ER expression [aOR, 0.91]). Our data suggest that ERBB2-low status alone should not influence neoadjuvant treatment decisions with currently approved regimens in this setting but could perhaps provide an incremental improvement in multivariable or multiomic models for pCR.^26^

We also found a slight improvement in OS in ERBB2-low breast cancer, particularly in advanced TNBC, although the size of the difference is of questionable clinical relevance, with overlapping survival curves when plotted by stage and receptor status. In a pooled analysis of 2310 patients receiving neoadjuvant chemotherapy, Denkert et al^15^ found a 5.9% improvement in OS in ERBB2-low TNBC, but a survival advantage was not seen in ERBB2-low hormone receptor–positive disease. However, most other studies have not found survival differences based on ERBB2-low status, highlighting that any prognostic association of ERBB2-low status is likely subtle.^5,11,27,28,29,30^ Undoubtedly, the introduction of antibody-drug conjugates will define the prognostic implications of ERBB2-low breast cancer moving forward given the marginal nature of these survival differences.

Whether there is a molecular basis that could explain these subtle differences in survival and chemotherapy responsiveness in ERBB2-low breast cancer remains to be ascertained. Prior studies have found that ERBB2-low cancers have an overrepresentation of the luminal A molecular subtype, which is known to have lower rates of pCR but maintains an excellent prognosis.^5,23^ Furthermore, ERBB2-low cases may be associated with the luminal androgen receptor (LAR) subtype of TNBC, given higher rates of androgen receptor positivity among ERBB2-low cases^28^ and high rates of ERBB2-enriched disease by 50-gene signature assay (PAM50) among patients with LAR TNBC.^31^ Because androgen receptor–positive TNBC has a better prognosis but worse response to chemotherapy, further study is needed to examine how much of the prognostic association of ERBB2-low TNBC is attributable to enrichment for the LAR subtype.^32^

The inaccuracies in IHC may further decrease the ability to accurately discern the length of survival or pCR differences associated with low-level ERBB2 expression and may have contributed to the inconsistent prognostic associations seen in the literature. More accurate quantification of levels of ERBB2 expression might aid in assessing associations with outcome. One criticism of using IHC for ERBB2 to define these tumors is that this assay was not designed to distinguish ERBB2-low from ERBB2-negative tumors but rather to distinguish the ERBB2-positive tumors that respond to traditional monoclonal antibodies such as trastuzumab.^21,33^ This is further complicated by the variability in ERBB2 scoring from institution to institution, reflected in the variability in proportions of ERBB2-low tumors from study to study^11,28,34^ and the lower rates of ERBB2-low diagnoses at academic centers seen in our study. Indeed, there is a pressing need to more precisely quantify low levels of ERBB2 expression to identify patients who might benefit from potent antibody-drug conjugates, as reflected in the DAISY trial,^35^ in which even patients classified as ERBB2-negative responded to T-DXd. Our analysis of *ERBB2* copy number and *ERBB2*/*CEP17* ratio did not offer promising discriminative ability for ERBB2-low disease. However, other surrogate molecular markers such as quantitative polymerase chain reaction or flow cytometry may help to more accurately identify candidates for ERBB2-directed antibody-drug conjugates.^36^

### Strengths and Limitations

This study has strengths, including the large patient cohort available for analysis, which is reflective of most cancer diagnoses across the US. This study has several important limitations that should be considered when interpreting these results. The NCDB includes only OS data, which limits associations of ERBB2-low status with cancer-specific prognosis, especially in the hormone receptor–positive cohort, where survival may lag years behind recurrence. Additionally, the NCDB lacks centralized assessment of ERBB2 status via IHC, perhaps highlighted by the different proportions of ERBB2-low disease in different institution types; therefore, some of the results may be associated with regional variation in practice of classifying cases as ERBB2 0 vs ERBB2 1+ as scored via IHC. Our correlation of ERBB2-low status with ISH results is limited by the fact that most patients with ERBB2 expression scored as 0 or 1+ via IHC did not have ISH performed and therefore must be interpreted with caution.

## Conclusions

The results of this cohort study suggest that there are associations between ERBB2 expression and hormone receptor expression and ductal tumor histological status but only slight differences in response to treatment and prognosis. These findings do not support classification of ERBB2-low breast cancer as a distinct clinical subtype. Further improvements are needed in molecular stratification of ERBB2 expression to understand the clinical significance of ERBB2-low breast cancer and identify patients who can benefit from novel therapies. Moving forward, the availability and use of ERBB2-directed antibody-drug conjugates will likely drive prognosis in ERBB2-low disease, rather than intrinsic differences in biology between ERBB2-low and ERBB2-negative breast cancer.

## Notes

Supplement 1. **eFigure 1.** CONSORT Diagram for Included Patients **eFigure 2.** HER2-Low Status by ER Expression and PR Expression **eFigure 3.** Density Plots of HER2/CEP17 Ratio and HER2 Copy Number for HER2-Low and HER2-0 Cancers **eTable 1.** Baseline Patient Demographics and Clinicopathologic Characteristics per HER2 Immunohistochemistry Results **eTable 2.** Sites of Metastatic Disease in HER2-0 Versus HER2-Low Cancers **eTable 3.** Baseline Patient Demographics and Clinicopathologic Characteristics of Patients Treated With Neoadjuvant Chemotherapy **eTable 4.** Overall Survival Versus HER2 IHC status, by Cancer Subtype and Stage **eTable 5.** Sensitivity and Specificity for Prediction of HER2-Low Status Using Select Cutoffs for HER2 ISH Results

Supplement 2.
Data Sharing Statement

## References

[1] Seshadri R, Firgaira FA, Horsfall DJ, McCaul K, Setlur V, Kitchen P; The South Australian Breast Cancer Study Group. Clinical significance of HER-2/neu oncogene amplification in primary breast cancer. J Clin Oncol. 1993;11(10):1936-1942. doi:10.1200/JCO.1993.11.10.1936 8105035

[2] Wolff AC, Hammond MEH, Allison KH, . Human epidermal growth factor receptor 2 testing in breast cancer: American Society of Clinical Oncology/College of American Pathologists clinical practice guideline focused update. Arch Pathol Lab Med. 2018;142(11):1364-1382. doi:10.5858/arpa.2018-0902-SA 29846104

[3] Fehrenbacher L, Cecchini RS, Geyer CE Jr, . NSABP B-47/NRG oncology phase III randomized trial comparing adjuvant chemotherapy with or without trastuzumab in high-risk invasive breast cancer negative for HER2 by FISH and with IHC 1+ or 2. J Clin Oncol. 2020;38(5):444-453. doi:10.1200/JCO.19.01455 31821109 PMC7007289

[4] Gianni L, Lladó A, Bianchi G, . Open-label, phase II, multicenter, randomized study of the efficacy and safety of two dose levels of pertuzumab, a human epidermal growth factor receptor 2 dimerization inhibitor, in patients with human epidermal growth factor receptor 2-negative metastatic breast cancer. J Clin Oncol. 2010;28(7):1131-1137. doi:10.1200/JCO.2009.24.1661 20124183 PMC4979215

[5] Schettini F, Chic N, Brasó-Maristany F, . Clinical, pathological, and PAM50 gene expression features of HER2-low breast cancer. NPJ Breast Cancer. 2021;7(1):1-13. doi:10.1038/s41523-020-00208-2 33397968 PMC7782714

[6] Tarantino P, Hamilton E, Tolaney SM, . HER2-low breast cancer: pathological and clinical landscape. J Clin Oncol. 2020;38(17):1951-1962. doi:10.1200/JCO.19.02488 32330069

[7] Schalper KA, Kumar S, Hui P, Rimm DL, Gershkovich P. A retrospective population-based comparison of HER2 immunohistochemistry and fluorescence in situ hybridization in breast carcinomas: impact of 2007 American Society of Clinical Oncology/College of American Pathologists criteria. Arch Pathol Lab Med. 2014;138(2):213-219. doi:10.5858/arpa.2012-0617-OA 24164555

[8] Modi S, Park H, Murthy RK, . Antitumor activity and safety of trastuzumab deruxtecan in patients with HER2-low-expressing advanced breast cancer: results from a phase Ib study. J Clin Oncol. 2020;38(17):1887-1896. doi:10.1200/JCO.19.02318 32058843 PMC7280051

[9] Modi S, Jacot W, Yamashita T, ; DESTINY-Breast04 Trial Investigators. Trastuzumab deruxtecan in previously treated HER2-low advanced breast cancer. N Engl J Med. 2022;387(1):9-20. doi:10.1056/NEJMoa2203690 35665782 PMC10561652

[10] Tarantino P, Jin Q, Tayob N, . Prognostic and biologic significance of ERBB2-low expression in early-stage breast cancer. JAMA Oncol. 2022;8(8):1177-1183. doi:10.1001/jamaoncol.2022.2286 35737367 PMC9227690

[11] Horisawa N, Adachi Y, Takatsuka D, . The frequency of low HER2 expression in breast cancer and a comparison of prognosis between patients with HER2-low and HER2-negative breast cancer by HR status. Breast Cancer. 2022;29(2):234-241. doi:10.1007/s12282-021-01303-3 34622383

[12] Guven DC, Kaya MB, Fedai B, . HER2-low breast cancer could be associated with an increased risk of brain metastasis. Int J Clin Oncol. 2022;27(2):332-339. doi:10.1007/s10147-021-02049-w 34661778

[13] Bao KKH, Sutanto L, Tse SSW, Man Cheung K, Chan JCH. The association of ERBB2-low expression with the efficacy of cyclin-dependent kinase 4/6 inhibitor in hormone receptor-positive, ERBB2-negative metastatic breast cancer. JAMA Netw Open. 2021;4(11):e2133132. doi:10.1001/jamanetworkopen.2021.33132 34739066 PMC8571658

[14] Mutai R, Barkan T, Moore A, . Prognostic impact of HER2-low expression in hormone receptor positive early breast cancer. Breast. 2021;60:62-69. doi:10.1016/j.breast.2021.08.016 34481367 PMC8414540

[15] Denkert C, Seither F, Schneeweiss A, . Clinical and molecular characteristics of HER2-low-positive breast cancer: pooled analysis of individual patient data from four prospective, neoadjuvant clinical trials. Lancet Oncol. 2021;22(8):1151-1161. doi:10.1016/S1470-2045(21)00301-6 34252375

[16] Bilimoria KY, Stewart AK, Winchester DP, Ko CY. The National Cancer Data Base: a powerful initiative to improve cancer care in the United States. Ann Surg Oncol. 2008;15(3):683-690. doi:10.1245/s10434-007-9747-3 18183467 PMC2234447

[17] von Elm E, Altman DG, Egger M, Pocock SJ, Gøtzsche PC, Vandenbroucke JP; STROBE Initiative. Strengthening the Reporting of Observational Studies in Epidemiology (STROBE) statement: guidelines for reporting observational studies. BMJ. 2007;335(7624):806-808. doi:10.1136/bmj.39335.541782.AD 17947786 PMC2034723

[18] White IR, Royston P, Wood AM. Multiple imputation using chained equations: issues and guidance for practice. Stat Med. 2011;30(4):377-399. doi:10.1002/sim.4067 21225900

[19] fmhoward/HER2Epidemiology. GitHub. Accessed September 3, 2022. https://github.com/fmhoward/HER2Epidemiology

[20] Tarantino P, Tayob N, Tolaney SM. Association of hormone receptors with clinical outcomes in patients with ERBB2-low breast cancer—reply. JAMA Oncol. 2023;9(1):147-148. doi:10.1001/jamaoncol.2022.5094 36327124

[21] Fernandez AI, Liu M, Bellizzi A, . Examination of low ERBB2 protein expression in breast cancer tissue. JAMA Oncol. 2022;8(4):1-4. doi:10.1001/jamaoncol.2021.7239 35113160 PMC8814969

[22] Kong X, Liu Z, Cheng R, . Variation in breast cancer subtype incidence and distribution by race/ethnicity in the United States from 2010 to 2015. JAMA Netw Open. 2020;3(10):e2020303. doi:10.1001/jamanetworkopen.2020.20303 33074325 PMC7573683

[23] Daly B, Olopade OI. A perfect storm: how tumor biology, genomics, and health care delivery patterns collide to create a racial survival disparity in breast cancer and proposed interventions for change. CA Cancer J Clin. 2015;65(3):221-238. doi:10.3322/caac.21271 25960198

[24] Howard FM, Olopade OI. Epidemiology of triple-negative breast cancer: a review. Cancer J. 2021;27(1):8-16. doi:10.1097/PPO.0000000000000500 33475288 PMC12050094

[25] Cappelletti V, Di Cosimo S, Pruneri G. Association of hormone receptors with clinical outcomes in patients with ERBB2-low breast cancer. JAMA Oncol. 2023;9(1):146-147. doi:10.1001/jamaoncol.2022.5091 36326737

[26] Sammut SJ, Crispin-Ortuzar M, Chin SF, . Multi-omic machine learning predictor of breast cancer therapy response. Nature. 2022;601(7894):623-629. doi:10.1038/s41586-021-04278-5 34875674 PMC8791834

[27] Agostinetto E, Rediti M, Fimereli D, . HER2-low breast cancer: molecular characteristics and prognosis. Cancers (Basel). 2021;13(11):2824. doi:10.3390/cancers13112824 34198891 PMC8201345

[28] Jacot W, Maran-Gonzalez A, Massol O, . Prognostic value of HER2-low expression in non-metastatic triple-negative breast cancer and correlation with other biomarkers. Cancers (Basel). 2021;13(23):6059. doi:10.3390/cancers13236059 34885167 PMC8656488

[29] de Moura Leite L, Cesca MG, Tavares MC, . HER2-low status and response to neoadjuvant chemotherapy in HER2 negative early breast cancer. Breast Cancer Res Treat. 2021;190(1):155-163. doi:10.1007/s10549-021-06365-7 34409551

[30] Gampenrieder SP, Rinnerthaler G, Tinchon C, . Landscape of HER2-low metastatic breast cancer (MBC): results from the Austrian AGMT_MBC-Registry. Breast Cancer Res. 2021;23(1):112. doi:10.1186/s13058-021-01492-x 34906198 PMC8670265

[31] Wang DY, Jiang Z, Ben-David Y, Woodgett JR, Zacksenhaus E. Molecular stratification within triple-negative breast cancer subtypes. Sci Rep. 2019;9(1):19107. doi:10.1038/s41598-019-55710-w 31836816 PMC6911070

[32] Gerratana L, Basile D, Buono G, . Androgen receptor in triple negative breast cancer: a potential target for the targetless subtype. Cancer Treat Rev. 2018;68:102-110. doi:10.1016/j.ctrv.2018.06.005 29940524

[33] Allison KH, Wolff AC. ERBB2-low breast cancer—is it a fact or fiction, and do we have the right assay? JAMA Oncol. 2022;8(4):610-611. doi:10.1001/jamaoncol.2021.7082 35113131

[34] Won HS, Ahn J, Kim Y, . Clinical significance of HER2-low expression in early breast cancer: a nationwide study from the Korean Breast Cancer Society. Breast Cancer Res. 2022;24(1):22. doi:10.1186/s13058-022-01519-x 35307014 PMC8935777

[35] Diéras V, Deluche E, Lusque A, . Abstract PD8-02: trastuzumab deruxtecan (T-DXd) for advanced breast cancer patients (ABC), regardless HER2 status: a phase II study with biomarkers analysis (DAISY). Cancer Res. 2022;82(4)(suppl):PD8-02. doi:10.1158/1538-7445.SABCS21-PD8-02

[36] Moutafi M, Robbins CJ, Yaghoobi V, . Quantitative measurement of HER2 expression to subclassify ERBB2 unamplified breast cancer. Lab Invest. 2022;102(10):1101-1108. Published online May 20, 2022. doi:10.1038/s41374-022-00804-9 35595825

