# Supplementary material for: Clinicopathologic Characteristics and Prognosis of ERBB2-Low Breast Cancer Among Patients in the National Cancer Database
Source: JAMA Oncol. 2023 Feb 23;9(4):500–10. doi: 10.1001/jamaoncol.2022.7476 (PMC9951099; doi:10.1001/jamaoncol.2022.7476)
Supplement: Supplement 1. — eFigure 1. CONSORT Diagram for Included Patients eFigure 2. HER2-Low Status by ER Expression and PR Expression eFigure 3. Density Plots of HER2/CEP17 Ratio and HER2 Copy Number for HER2-Low and HER2-0 Cancers eTable 1. Baseline Patient Demographics and Clinicopathologic Characteristics per HER2 Immunohistochemistry Results eTable 2. Sites of Metastatic Disease in HER2-0 Versus HER2-Low Cancers eTable 3. Baseline Patient Demographics and Clinicopathologic Characteristics of Patients Treated With Neoadjuvant Chemotherapy eTable 4. Overall Survival Versus HER2 IHC status, by Cancer Subtype and Stage eTable 5. Sensitivity and Specificity for Prediction of HER2-Low Status Using Select Cutoffs for HER2 ISH Results [file jamaoncol-e227476-s001.pdf]

Supplementary Online Content

Peiffer DS, Zhao F, Chen N, et al. Clinicopathologic characteristics and prognosis of ERBB2-low breast cancer among patients in the National Cancer Database. *JAMA Oncol.* Published online February 23, 2023. doi:10.1001/jamaoncol.2022.7476

eFigure 1. CONSORT Diagram for Included Patients

eFigure 2. ERBB2-Low Status by ER Expression and PR Expression

eFigure 3. Density Plots of ERBB2/CEP17 Ratio and ERBB2 Copy Number for ERBB2-Low and ERBB2-Negative Cancers

eTable 1. Baseline Patient Demographics and Clinicopathologic Characteristics per ERBB2 Immunohistochemistry Results

eTable 2. Sites of Metastatic Disease in ERBB2-Negative Versus ERBB2-Low cancers

eTable 3. Baseline Patient Demographics and Clinicopathologic Characteristics of Patients Treated With Neoadjuvant Chemotherapy

eTable 4. Overall Survival Versus ERBB2 Immunohistochemistry Results, by Cancer Subtype and Stage

eTable 5. Sensitivity and Specificity for Prediction of ERBB2-Low Status Using Select Cutoffs for ERBB2 ISH Results

This supplementary material has been provided by the authors to give readers additional information about their work.

© 2023 Peiffer DS et al. *JAMA Oncology*.

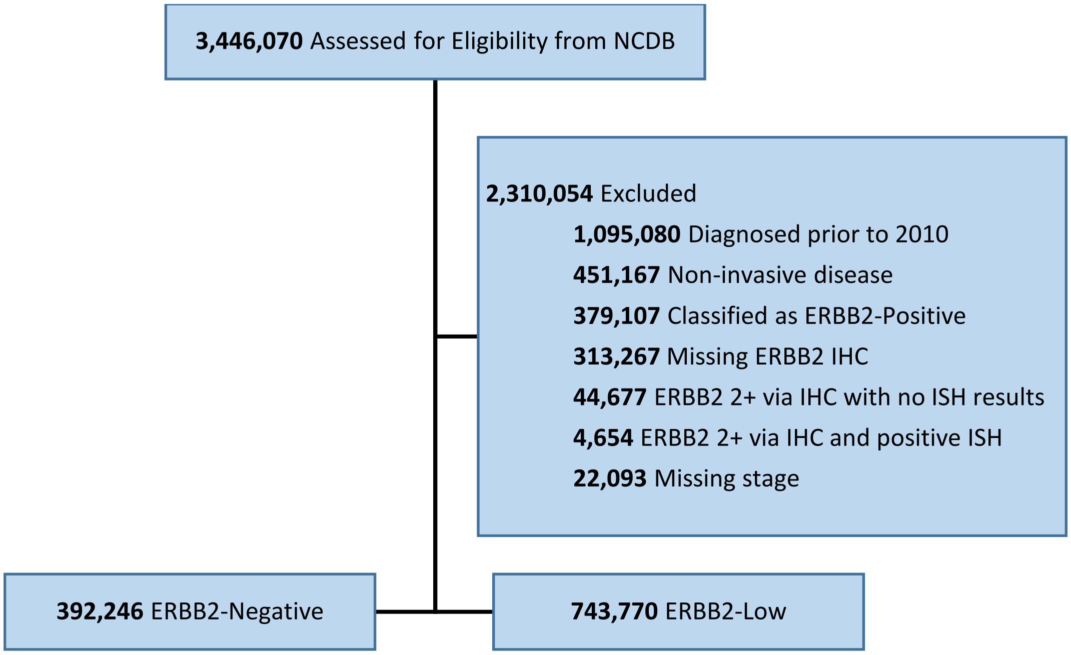

eFigure 1. CONSORT Diagram for Included Patients. Abbreviations: NCDB = National Cancer Database. ERBB2 = erb-b2 receptor tyrosine kinase 2. IHC = Immunohistochemistry. ISH = in situ hybridization.

© 2023 Peiffer DS et al. *JAMA Oncology*.

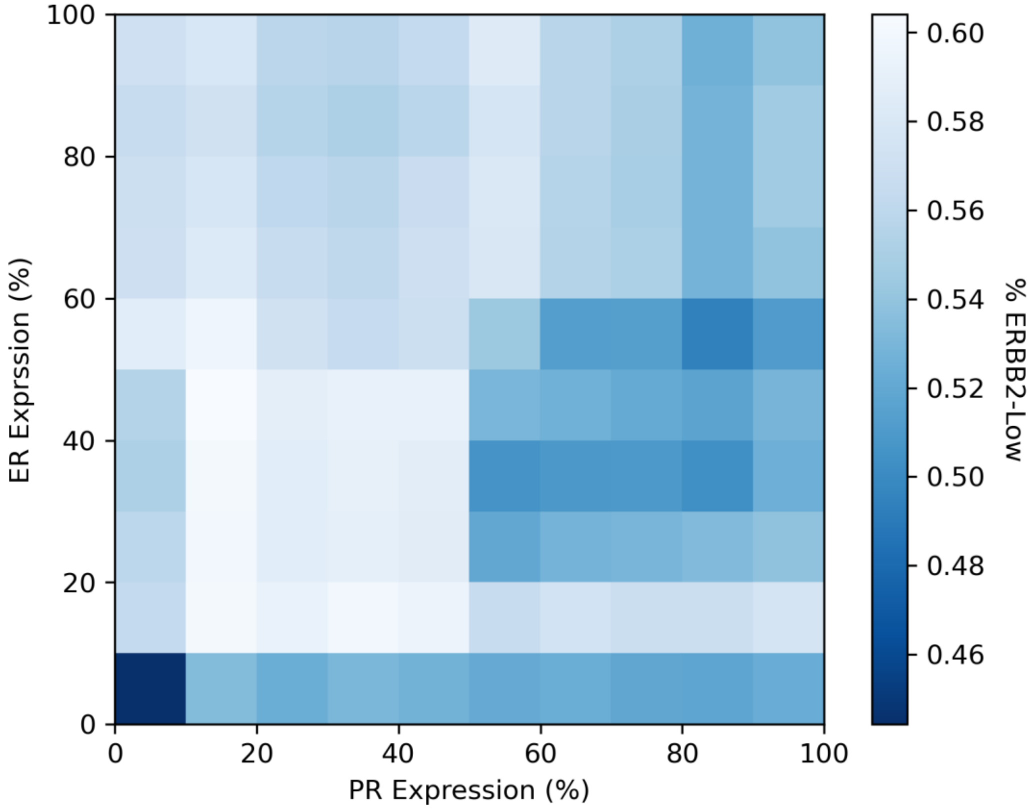

eFigure 2. ERBB2-Low Status by ER Expression and PR Expression. Results from 274,716 patients with percentage ER and PR expression available. Patients with weak ER expression demonstrate a decrease in rates of ERBB2-low disease when PR expression is strong (bottom right) as opposed to weak (bottom left). Abbreviations: ERBB2 = erb-b2 receptor tyrosine kinase 2. ER = estrogen receptor. PR = progesterone receptor.

© 2023 Peiffer DS et al. *JAMA Oncology*.

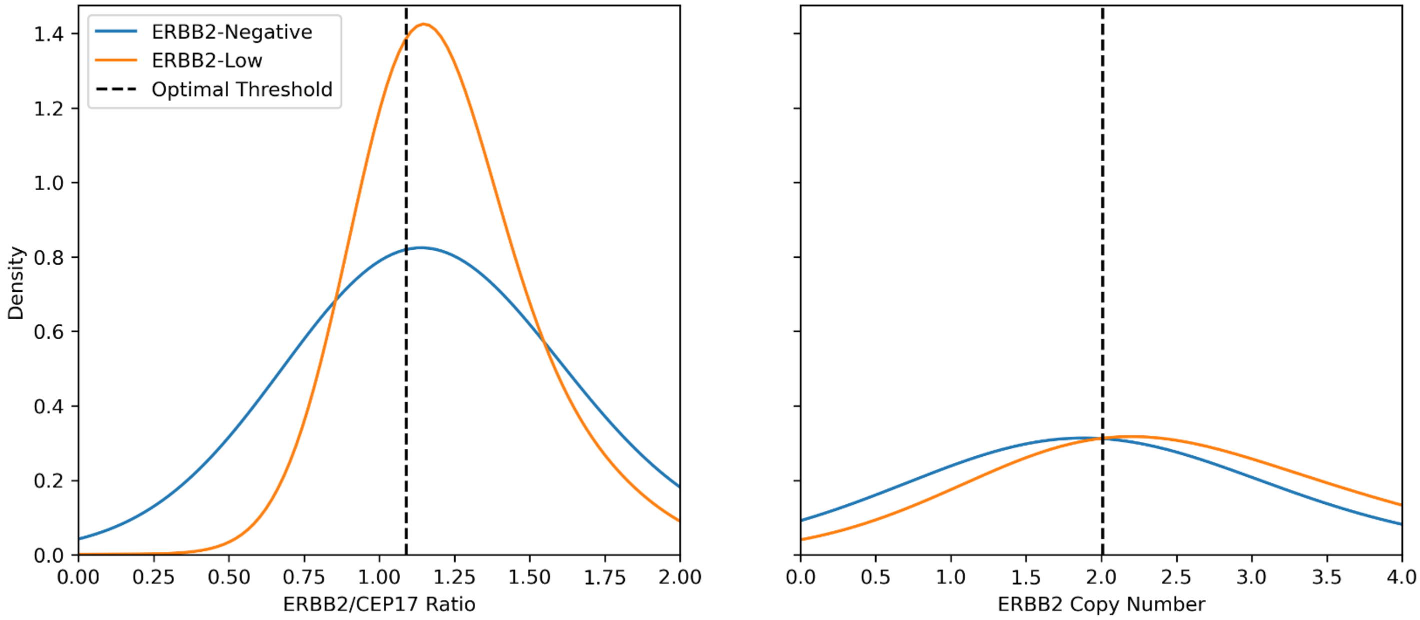

eFigure 3. Density Plots of ERBB2/CEP17 Ratio and ERBB2 Copy Number for ERBB2-Low and ERBB2-Negative Cancers. A total of n = 74,035 patients had dual probe ERBB2/CEP17 ratio results (**left**), and a threshold ratio of 1.09 optimized Youden's index, achieving a sensitivity of 77% and specificity of 37% for ERBB2-low disease. Similarly, n = 99,541 had ERBB2 copy number results (**right**), and a threshold of 2.01 had a sensitivity of 61% and specificity of 58% for identifying ERBB2-low disease. Abbreviations: ERBB2 = erb-b2 receptor tyrosine kinase 2.

© 2023 Peiffer DS et al. *JAMA Oncology*.

eTable 1. Baseline Patient Demographics and Clinicopathologic Characteristics per ERBB2 Immunohistochemistry Results. P-values are listed for a chi-squared test for categorical variables and a two-sided t-test for continuous variables. Abbreviations: ERBB2 = erb-b2 receptor tyrosine kinase 2. SD = standard deviation. ER = estrogen receptor. PR = progesterone receptor.

		Overall	ERBB2 0	ERBB2 1+	ERBB2 2+	p-Value
n		1136016	392246	548293	195477	
Age, n (%)	< 40	46482 (4.1)	17581 (4.5)	20620 (3.8)	8281 (4.2)	<0.001
	40 - 49	158040 (13.9)	55541 (14.2)	74976 (13.7)	27523 (14.1)	
	50 - 59	255811 (22.5)	88438 (22.5)	123304 (22.5)	44069 (22.5)	
	60 - 69	326651 (28.8)	111515 (28.4)	159406 (29.1)	55730 (28.5)	
	70 - 79	237176 (20.9)	81558 (20.8)	115678 (21.1)	39940 (20.4)	
	≥ 80	111856 (9.8)	37613 (9.6)	54309 (9.9)	19934 (10.2)	
Sex, n (%)	Female	1125645 (99.1)	389318 (99.3)	543013 (99.0)	193314 (98.9)	<0.001
	Male	10371 (0.9)	2928 (0.7)	5280 (1.0)	2163 (1.1)	
Race / Ethnicity, n (%)	Asian	41619 (3.7)	14112 (3.6)	19473 (3.6)	8034 (4.1)	<0.001
	Hispanic	62985 (5.6)	24536 (6.3)	27978 (5.1)	10471 (5.4)	
	Native American	3242 (0.3)	972 (0.2)	1634 (0.3)	636 (0.3)	
	Non-Hispanic Black	126977 (11.3)	47194 (12.1)	58236 (10.7)	21547 (11.1)	
	Non-Hispanic White	886131 (78.6)	299961 (77.1)	433984 (79.8)	152186 (78.5)	
	Other	5737 (0.5)	2217 (0.6)	2614 (0.5)	906 (0.5)	
Facility Type, n (%)	Academic / Research	333794 (30.6)	127586 (34.1)	150347 (28.5)	55861 (29.8)	<0.001
	Community Cancer Program	78218 (7.2)	26174 (7.0)	39368 (7.5)	12676 (6.8)	
	Comprehensive Community Cancer Program	450549 (41.4)	146217 (39.0)	225667 (42.8)	78665 (42.0)	
	Integrated Network Cancer Program	226973 (20.8)	74688 (19.9)	112291 (21.3)	39994 (21.4)	
Charlson/Deyo Score, n (%)	0	931623 (82.0)	322257 (82.2)	449731 (82.0)	159635 (81.7)	<0.001
	>= 1	204393 (18.0)	69989 (17.8)	98562 (18.0)	35842 (18.3)	
Grade, n (%)	1	284050 (26.1)	91339 (24.4)	151338 (28.8)	41373 (22.0)	<0.001
	2	514099 (47.3)	167171 (44.6)	250798 (47.8)	96130 (51.2)	
	3	289022 (26.6)	115949 (31.0)	122660 (23.4)	50413 (26.8)	
Histologic Subtype, n (%)	Ductal	883090 (77.7)	296283 (75.5)	427789 (78.0)	159018 (81.3)	<0.001
	Ductal and Lobular	60000 (5.3)	20430 (5.2)	29711 (5.4)	9859 (5.0)	
	Inflammatory	1758 (0.2)	677 (0.2)	760 (0.1)	321 (0.2)	
	Lobular	132380 (11.7)	48657 (12.4)	64483 (11.8)	19240 (9.8)	
	Medullary	1461 (0.1)	734 (0.2)	571 (0.1)	156 (0.1)	
	Metaplastic	6236 (0.5)	3989 (1.0)	1789 (0.3)	458 (0.2)	
	Mucinous	22457 (2.0)	10410 (2.7)	9695 (1.8)	2352 (1.2)	
	Others	18347 (1.6)	7706 (2.0)	7839 (1.4)	2802 (1.4)	
	Paget Disease	610 (0.1)	181 (0.0)	288 (0.1)	141 (0.1)	
	Papillary	3049 (0.3)	1057 (0.3)	1551 (0.3)	441 (0.2)	
	Sarcoma	230 (0.0)	156 (0.0)	60 (0.0)	14 (0.0)	
	Tubular	6398 (0.6)	1966 (0.5)	3757 (0.7)	675 (0.3)	
T Stage, n (%)	0	2924 (0.3)	1165 (0.3)	1346 (0.3)	413 (0.2)	<0.001
	1	677942 (65.2)	230180 (64.3)	336525 (66.9)	111237 (62.5)	
	2	284172 (27.3)	99427 (27.8)	131739 (26.2)	53006 (29.8)	
	3	52152 (5.0)	19408 (5.4)	23600 (4.7)	9144 (5.1)	
	4	21914 (2.1)	7857 (2.2)	9803 (1.9)	4254 (2.4)	
N Stage, n (%)	0	723240 (72.2)	251448 (72.8)	351896 (72.6)	119896 (69.8)	<0.001
	1	216005 (21.6)	72358 (21.0)	103481 (21.3)	40166 (23.4)	
	2	40267 (4.0)	13564 (3.9)	19078 (3.9)	7625 (4.4)	
	3	22208 (2.2)	7812 (2.3)	10252 (2.1)	4144 (2.4)	
Stage Group, n (%)	I	678389 (59.7)	230618 (58.8)	335842 (61.3)	111929 (57.3)	<0.001
	II	309228 (27.2)	107907 (27.5)	144990 (26.4)	56331 (28.8)	
	III	98683 (8.7)	35646 (9.1)	44789 (8.2)	18248 (9.3)	
	IV	49716 (4.4)	18075 (4.6)	22672 (4.1)	8969 (4.6)	
Bone Mets at Diagnosis, n (%)	No	1097880 (97.7)	378662 (97.7)	530617 (97.7)	188601 (97.5)	<0.001
	Yes	26141 (2.3)	8899 (2.3)	12389 (2.3)	4853 (2.5)	
Brain Mets at Diagnosis, n (%)	No	1121087 (99.8)	386395 (99.7)	541719 (99.8)	192973 (99.8)	<0.001
	Yes	2575 (0.2)	1024 (0.3)	1143 (0.2)	408 (0.2)	
Liver Mets at Diagnosis, n (%)	No	1116283 (99.3)	384631 (99.3)	539588 (99.4)	192064 (99.3)	<0.001
	Yes	7463 (0.7)	2849 (0.7)	3291 (0.6)	1323 (0.7)	
Lung Mets at Diagnosis, n (%)	No	1112823 (99.0)	383552 (99.0)	537906 (99.1)	191365 (99.0)	<0.001
	Yes	10790 (1.0)	3882 (1.0)	4918 (0.9)	1990 (1.0)	
ERBB2/CEP17 Ratio, mean (SD)		1.3 (1.3)	1.3 (2.2)	1.3 (1.6)	1.3 (0.6)	0.001
ERBB2 Copies, mean (SD)		2.7 (4.2)	2.4 (4.7)	2.6 (4.5)	2.9 (3.7)	<0.001
Receptor Status, n (%)	ER+PR+	856039 (75.8)	269903 (69.2)	434346 (79.7)	151790 (78.2)	<0.001
	ER+PR-	109383 (9.7)	38144 (9.8)	50464 (9.3)	20775 (10.7)	

© 2023 Peiffer DS et al. *JAMA Oncology*.

	**ER-PR+**	9559 (0.8)	4201 (1.1)	4001 (0.7)	1357 (0.7)	
	**ER-PR-**	153818 (13.6)	77693 (19.9)	55901 (10.3)	20224 (10.4)	
**ER (% Positive), mean (SD)**		78.1 (35.2)	70.8 (40.2)	82.7 (30.8)	82.0 (31.9)	<0.001
**PR (% Positive), mean (SD)**		53.9 (40.8)	49.8 (42.2)	57.9 (39.6)	52.4 (39.9)	<0.001
**Ki67 (% Positive), mean (SD)**		24.6 (26.8)	27.6 (30.1)	22.3 (25.2)	24.1 (22.4)	<0.001
**OncotypeDx Score, n (%)**	**High (26+)**	35287 (12.5)	10734 (12.4)	16594 (11.7)	7959 (14.7)	<0.001
	**Intermediate (11 - 25)**	176469 (62.5)	53496 (61.6)	88775 (62.7)	34198 (63.3)	
	**Low (0 - 10)**	70673 (25.0)	22675 (26.1)	36127 (25.5)	11871 (22.0)	
**Chemotherapy, n (%)**	**Chemotherapy**	386952 (34.5)	144168 (37.2)	172816 (31.9)	69968 (36.2)	<0.001
	**No Chemo**	735429 (65.5)	243244 (62.8)	368948 (68.1)	123237 (63.8)	
**Hormonal Therapy, n (%)**	**Hormonal Therapy**	813107 (73.3)	258075 (67.4)	407531 (76.1)	147501 (77.2)	<0.001
	**No Hormonal Therapy**	296554 (26.7)	124931 (32.6)	127954 (23.9)	43669 (22.8)	

© 2023 Peiffer DS et al. *JAMA Oncology*.

eTable 2. Sites of Metastatic Disease in ERBB2-Negative Versus ERBB2-Low cancers. Adjusted odds ratio is listed for a multivariable logistic regression including age, sex, comorbidity score, facility type, race / ethnicity, grade, histologic subtype, and quantitative receptor status for prediction of presence of metastasis at diagnosis for indicated site among patients with Stage IV disease. Imputation was used for missing values. Abbreviations: ERBB2 = erb-b2 receptor tyrosine kinase 2.

Metastatic Site	n ERBB2-Negative	n ERBB2-Low	Adjusted OR (95% CI)	p
Brain	13,248	24,114	0.88 (0.81 - 0.96)	0.002
Lung	13,263	24,050	1.00 (0.95 – 1.05)	0.93
Liver	13,309	24,137	0.95 (0.90 – 1.00)	0.06
Bone	13,390	24,331	1.02 (0.97 – 1.07)	0.35

© 2023 Peiffer DS et al. *JAMA Oncology*.

eTable 3. Baseline Patient Demographics and Clinicopathologic Characteristics of Patients Treated With Neoadjuvant Chemotherapy. P-values are listed for a chi-squared test for categorical variables and a two-sided t-test for continuous variables. Abbreviations: ERBB2 = erb-b2 receptor tyrosine kinase 2. SD = standard deviation. ER = estrogen receptor. PR = progesterone receptor.

		Missing	Overall	ERBB2-Negative	ERBB2-Low	p-Value
n			109588	44019	65569	
Age, n (%)	< 40	0	15665 (14.3)	6716 (15.3)	8949 (13.6)	<0.001
	40 - 49		26946 (24.6)	11098 (25.2)	15848 (24.2)	
	50 - 59		31534 (28.8)	12487 (28.4)	19047 (29.0)	
	60 - 69		24871 (22.7)	9622 (21.9)	15249 (23.3)	
	70 - 79		9314 (8.5)	3601 (8.2)	5713 (8.7)	
	≥ 80		1258 (1.1)	495 (1.1)	763 (1.2)	
Sex, n (%)	Female	0	109003 (99.5)	43829 (99.6)	65174 (99.4)	<0.001
	Male		585 (0.5)	190 (0.4)	395 (0.6)	
Race / Ethnicity, n (%)	Asian	874	4531 (4.2)	1721 (3.9)	2810 (4.3)	<0.001
	Hispanic		9532 (8.8)	4126 (9.4)	5406 (8.3)	
	Native American		390 (0.4)	126 (0.3)	264 (0.4)	
	Non-Hispanic Black		20292 (18.7)	8649 (19.8)	11643 (17.9)	
	Non-Hispanic White		73274 (67.4)	28752 (65.8)	44522 (68.5)	
	Other		695 (0.6)	302 (0.7)	393 (0.6)	
Facility Type, n (%)	Academic / Research	15665	32507 (34.6)	13672 (36.7)	18835 (33.3)	<0.001
	Community Cancer Program		5519 (5.9)	2201 (5.9)	3318 (5.9)	
	Comprehensive Community Cancer Program		35597 (37.9)	13514 (36.2)	22083 (39.0)	
	Integrated Network Cancer Program		20300 (21.6)	7916 (21.2)	12384 (21.9)	
Charlson/Deyo Score, n (%)	0	0	94226 (86.0)	37855 (86.0)	56371 (86.0)	0.914
	>= 1		15362 (14.0)	6164 (14.0)	9198 (14.0)	
Grade, n (%)	1	5645	6051 (5.8)	1929 (4.6)	4122 (6.6)	<0.001
	2		34191 (32.9)	11269 (27.0)	22922 (36.9)	
	3		63701 (61.3)	28599 (68.4)	35102 (56.5)	
Histologic Subtype, n (%)	Ductal	0	93344 (85.2)	37657 (85.5)	55687 (84.9)	<0.001
	Ductal and Lobular		3765 (3.4)	1343 (3.1)	2422 (3.7)	
	Inflammatory		821 (0.7)	331 (0.8)	490 (0.7)	
	Lobular		7512 (6.9)	2651 (6.0)	4861 (7.4)	
	Medullary		135 (0.1)	72 (0.2)	63 (0.1)	
	Metaplastic		1264 (1.2)	763 (1.7)	501 (0.8)	
	Mucinous		409 (0.4)	128 (0.3)	281 (0.4)	
	Others		2137 (2.0)	1004 (2.3)	1133 (1.7)	
	Paget Disease		41 (0.0)	12 (0.0)	29 (0.0)	
	Papillary		99 (0.1)	25 (0.1)	74 (0.1)	
	Sarcoma		30 (0.0)	20 (0.0)	10 (0.0)	
	Tubular		31 (0.0)	13 (0.0)	18 (0.0)	
T Stage, n (%)	0	1601	442 (0.4)	200 (0.5)	242 (0.4)	<0.001
	1		20535 (19.0)	8527 (19.7)	12008 (18.6)	
	2		54626 (50.6)	22248 (51.3)	32378 (50.1)	
	3		20562 (19.0)	7913 (18.3)	12649 (19.6)	
	4		11822 (10.9)	4468 (10.3)	7354 (11.4)	
N Stage, n (%)	0	1510	45746 (42.3)	19833 (45.7)	25913 (40.1)	<0.001
	1		49153 (45.5)	18424 (42.4)	30729 (47.5)	
	2		7614 (7.0)	3028 (7.0)	4586 (7.1)	
	3		5565 (5.1)	2138 (4.9)	3427 (5.3)	
Stage Group, n (%)	I	0	13853 (12.6)	5858 (13.3)	7995 (12.2)	<0.001
	II		60115 (54.9)	24001 (54.5)	36114 (55.1)	
	III		35620 (32.5)	14160 (32.2)	21460 (32.7)	
ERBB2/CEP17 Ratio, mean (SD)		73933	1.3 (1.3)	1.3 (2.2)	1.3 (1.0)	0.569
ERBB2 Copies, mean (SD)		99407	2.8 (3.7)	2.5 (4.1)	2.9 (3.5)	<0.001
Receptor Status, n (%)	ER+PR+	570	46742 (42.9)	13851 (31.6)	32891 (50.4)	<0.001
	ER+PR-		12498 (11.5)	4448 (10.2)	8050 (12.3)	
	ER-PR+		2636 (2.4)	1145 (2.6)	1491 (2.3)	
	ER-PR-		47142 (43.2)	24361 (55.6)	22781 (34.9)	
ER (% Positive), mean (SD)		77646	39.4 (45.1)	27.9 (41.5)	49.0 (45.7)	<0.001
PR (% Positive), mean (SD)		77649	24.0 (36.6)	17.2 (32.7)	29.7 (38.6)	<0.001
Ki67 (% Positive), mean (SD)		92957	49.8 (34.8)	55.0 (37.8)	45.5 (31.5)	<0.001
OncotypeDx Score, n (%)	High (26+)	107025	1100 (42.9)	327 (44.2)	773 (42.4)	0.140
	Intermediate (11 - 25)		1243 (48.5)	339 (45.9)	904 (49.6)	
	Low (0 - 10)		220 (8.6)	73 (9.9)	147 (8.1)	
Hormonal Therapy, n (%)	Hormonal Therapy	1510	55228 (51.1)	17173 (39.6)	38055 (58.9)	<0.001
	No Hormonal Therapy		52850 (48.9)	26242 (60.4)	26608 (41.1)	
Pathologic Response	Residual Disease	2026	80599 (80.8)	30316 (76.4)	50283 (83.7)	<0.001
	Pathologic Complete Response		19184 (19.2)	9372 (23.6)	9812 (16.3)	

© 2023 Peiffer DS et al. *JAMA Oncology*.

eTable 4. Overall Survival Versus ERBB2 Immunohistochemistry Results, by Cancer Subtype and Stage. Adjusted hazard ratio is listed for a multivariable Cox proportional hazards model including age, sex comorbidity score, facility type, race / ethnicity, grade, histologic subtype, and quantitative estrogen and progesterone receptor status in the hormone receptor positive model, with imputation used for missing values. Abbreviations: ERBB2 = erb-b2 receptor tyrosine kinase 2. HR = hazard ratio. CI = confidence interval.

**Triple Negative Breast Cancer**							
**Stage**	**n ERBB2 0**	**n ERBB2 1+**	**n ERBB2 2+**	**Adjusted HR, ERBB2 1+ (95% CI)**	**p**	**Adjusted HR, ERBB2 2+ (95% CI)**	**p**
1	22662	17152	6185	1.02 (0.98 - 1.06)	0.40	0.98 (0.93 - 1.04)	0.54
2	23467	17911	6358	0.97 (0.94 - 1.0)	0.03	0.89 (0.85 – 0.93)	< 0.001
3	8021	6281	2436	0.96 (0.92 - 1.00)	0.06	0.86 (0.81 - 0.91)	< 0.001
4	3067	2237	791	0.94 (0.89 – 0.98)	0.009	0.87 (0.81 – 0.93)	< 0.001
**Hormone Receptor Positive**							
1	154377	246550	80685	1.01 (0.99 - 1.02)	0.27	1.00 (0.99 - 1.02)	0.78
2	63386	103504	40215	1.0 (0.98 - 1.02)	0.87	0.98 (0.96 - 1.01)	0.16
3	17998	28893	11637	0.98 (0.96 - 1.01)	0.21	0.96 (0.93 – 0.99)	0.01
4	8040	12726	5141	0.98 (0.96 - 1.01)	0.20	0.93 (0.90 - 0.97)	< 0.001

© 2023 Peiffer DS et al. *JAMA Oncology*.

eTable 5. Sensitivity and Specificity for Prediction of ERBB2-Low Status Using Select Cutoffs for ERBB2 ISH Results. Results are computed using n = 74,035 patients had dual probe ERBB2/CEP17 ratio results and n = 99,541 who had ERBB2 copy number results. Abbreviations: ERBB2 = erb-b2 receptor tyrosine kinase 2. ISH = in situ hybridization.

ERBB2 Copy Number			ERBB2/CEP17 Ratio		
Cutoff	Sensitivity (%)	Specificity (%)	Cutoff	Sensitivity (%)	Specificity (%)
1	98.8	4.1	1	98.7	3.3
1.15	92.1	18.9	1.05	79.2	33.8
1.3	89	23	1.1	76	37.4
1.45	85.7	27.3	1.15	52.6	58.6
1.6	84.1	29.6	1.2	50.2	61.2
1.75	79.3	37	1.25	34.1	74.5
1.9	74.7	42.6	1.3	32.4	76.1
2.05	61.1	58.2	1.35	22.6	83.7
2.2	55.4	63.5	1.4	21.6	84.5
2.35	46.7	71.8	1.45	15.5	88.6
2.5	43.1	75.1	1.5	14.9	89.1
2.65	36.8	80.5	1.55	10.8	91.8
2.8	34.1	82.4	1.6	10.3	92.1
2.95	29	86.1	1.65	7.4	93.9
3.1	26.7	87.6	1.7	7.1	94.2
3.25	22.6	89.8	1.75	4.7	95.5
3.4	20.8	90.7	1.8	4.5	95.7
3.55	16.9	92.4	1.85	2.6	97.1
3.7	15	93.3	1.9	2.5	97.2
3.85	11.2	94.6	1.95	1.6	97.9
4	9.6	95.4	2	1.5	98

© 2023 Peiffer DS et al. *JAMA Oncology*.
