# Supplementary material for: Clinicopathologic Characteristics and Prognosis of ERBB2-Low Breast Cancer Among Patients in the National Cancer Database
Source: JAMA Oncol. 2023 Feb 23;9(4):500–10. doi: 10.1001/jamaoncol.2022.7476 (PMC9951099; doi:10.1001/jamaoncol.2022.7476)
Supplement: Supplement 2. — Data Sharing Statement [file jamaoncol-e227476-s002.pdf]

Data Sharing Statement

Peiffer. Demographic and Clinicopathological Characteristics and Prognosis of ERBB2-Low Breast Cancer Among Patients in the National Cancer Database. *JAMA Oncol*. Published February 23, 2023. doi:10.1001/jamaoncol.2022.7476

Data
Data available: No

Additional Information
Explanation for why data not available: The data collected for this study is maintained by the American Cancer Society / American College of Surgeons and can only be obtained through a direct request to those organizations.
